# ‘Roll-your-own’ cigarette smoking in South Africa between 2007 and 2010

**DOI:** 10.1186/1471-2458-13-597

**Published:** 2013-06-24

**Authors:** Olalekan A Ayo-Yusuf, Bukola G Olutola

**Affiliations:** 1 Department of Community Dentistry, Faculty of Health Sciences, University of Pretoria, Pretoria, South Africa; 2 School of Health Systems and Public Health, University of Pretoria, Pretoria, South Africa

**Keywords:** Smoking, Roll-your-own cigarettes, Factory-made cigarettes, South Africa, Self-efficacy, Quitting

## Abstract

**Background:**

The prevalence of smoking and consumption of cigarettes have decreased in South Africa over the last 20 years. This decrease is a result of comprehensive tobacco control legislation, particularly large cigarette tax increases. However, little attention has been given to the potential use of ‘roll-your-own’ cigarettes as cheaper alternatives, especially among the socio-economically disadvantaged population. This study therefore sought to determine socio-demographic correlates of ‘roll-your-own’ cigarette use among South African adults (2007–2010).

**Methods:**

This secondary data analysis used a merged dataset from two nationally representative samples of 2 907 and 3 112 South African adults (aged ≥16 years) who participated in the 2007 and 2010 annual South African Social Attitude Surveys respectively. The surveys used a face-to-face interviewer-administered questionnaire. The overall response rates were 83.1% for 2007 and 88.9% for 2010. Data elicited included socio-demographic data, current smoking status, type of tobacco products used, past quit attempts and self-efficacy in quitting. Data analysis included chi-square statistics and multi-variable adjusted logistic regression analysis.

**Results:**

Of the 1 296 current smokers in this study, 24.1% (n = 306) reported using roll-your-own cigarettes. Some of whom also smoked factory-made cigarettes. Roll-your-own cigarette smoking was most common among black Africans and was more common among male smokers than among female smokers (27% vs 15.8%; p < 0.01). Compared to smokers who exclusively used factory-made cigarettes, roll-your-own cigarette smokers were less confident that they could quit, more likely to be less educated, and more likely to reside in rural areas. The odds of use of roll-your-own cigarette were significantly higher in 2010 than in 2007 (OR = 1.24; 95% CI: 1.07-1.44).

**Conclusions:**

Despite an aggregate decline in smoking prevalence, roll-your-own cigarette smoking has increased and is particularly common among smokers in the lower socio-economic group. The findings also suggest the need for a more intensive treatment intervention to increase self-efficacy to quit among roll-your-own cigarette smokers.

## Background

In South Africa, smoking prevalence has been reported to have decreased from 34% in 1993 to 21.4% in 2003 [1]. This decrease has been attributed to an increase in the price of factory-made cigarettes, anti-smoking legislation and greater public awareness [2]. Between 1994 and 1999, the real excise cigarette taxes increased by up to 149% [3]. However, the public health impact of cigarette taxes may be lessened if, instead of quitting smokers switch to roll-your-own cigarettes, which usually cost less than factory-made cigarettes [1]. Such switching between products is particularly likely in South Africa because the tax structure for tobacco products provides an incentive for smokers to substitute roll-your-own cigarettes for factory-made cigarettes. For example, in South Africa, the 2011/2012 excise tax on cigarette tobacco was 210.51 South African Rands (R) per kilogram, while the excise tax on the pipe tobacco used for roll-your-own cigarettes was R119.16 per kilogram in packages weighing less than 5 kg [4].

Although some smokers regard roll-your-own cigarettes as a safer alternative, roll-your-own cigarette smokers have actually been reported to be more likely to be exposed to higher levels of smoke constituents than smokers of factory-made cigarettes [5,6]. The manufacturers of factory-made cigarettes control the weight, diameter, packing density of the tobacco and the porosity of the wrapping paper used in their products. This is not the case for roll-your-own cigarettes, for which these elements are controlled by the user, thereby giving rise to wide variation in the finished products [7]. Roll-your-own cigarette smokers may take more puffs, inhale more smoke per cigarette and for longer periods [6] and have been reported to be less likely to make quit attempts than those who smoke factory-made cigarettes [8,9].

In South Africa, roll-your-own cigarettes are commonly made from pipe tobacco available in different pack sizes; a 5 g pack is the smallest and the most popular [unpublished data]. Unlike in resource-rich countries [6,10], roll-your-own cigarette tobacco in South Africa is predominantly smoked rolled in strips of newspaper without filters, thus making the cigarettes potentially more dangerous [1]. However, thus far, little attention has been given to the use of roll-your-own cigarettes in South Africa. This study therefore sought to determine socio-demographic correlates of roll-your-own cigarette use among South African adults between 2007 and 2010.

## Methods

This secondary data analysis used data obtained from two nationally representative samples of South African adults (aged ≥ 16 years) who were non-institutionalized and participated in the 2007 (n = 2 907) and 2010 (n = 3 112) annual South African Social Attitude Survey (SASAS). The SASAS is a household survey which uses a multi-stage probability sampling strategy with census enumeration areas as the primary sampling unit. For each of the two years, a sample of 3 500 households was drawn from the master sample of the South African Human Sciences Research Council. This SASAS sample was stratified by socio-demographic domain for each province and geographical subtypes, namely tribal areas, formal rural, formal urban and informal urban. This stratification is designed to ensure sufficient geographical distribution across all nine provinces, and adequate distribution between South Africa’s four race groups.^a^ From each of the households, one eligible person (≥16 years old) was randomly selected for participation in the survey. The overall response rates were 83.1% for the 2007 survey and 88.9% for 2010 survey.

### Measures

The SASAS uses a face-to-face interviewer-administered questionnaire to obtain information on socio-demographic data such as age, gender, race and socio-economic status. In both datasets, participants were also asked: ‘Do you use or have you used any of the following products in the past?’ The tobacco products that were listed included factory-made cigarettes and roll-your-own cigarettes. Smokers were also asked to indicate frequency of use. The roll-your-own cigarette smokers and factory-made cigarette smokers who responded ‘Every day’ and ‘Some days’ with regard to their use of the corresponding products were regarded as current smokers. A similar approach was used to categorize current snuff users, irrespective of whether respondents reported using nasal or oral snuff. It was assumed that those who did not indicate that they were planning to quit smoking within the next month or next 6 months or some time in future, beyond 6 months, have no intention of quitting. Self-efficacy in quitting was assessed by asking the question ‘If you tried to stop, how likely do you think it is that you would succeed in giving up smoking?’ The options were (1) ‘very likely’, (2) ‘fairly likely’, (3) ‘Not very likely’ and (4) ‘Not at all likely’. Other information obtained using questions similar to those used in previously published studies [5,11,12], included the type(s) of tobacco product used, any past quit attempts, ever being advised to quit smoking by a health professional, belief about the harmfulness of second-hand smoke, and rules about smoking in the workplace and in the home. The datasets from the two surveys were then merged (N = 6 019).

### Statistical analyses

The data were analysed using STATA Release 10 (Stata Corporation, College Station, Texas, USA), with appropriate weighting of selection probabilities and taking into consideration the complex sample design used in the SASAS. Group differences were assessed using chi-square statistics. Statistical comparisons were made between the categories of factory-made cigarette smoking and roll-your-own cigarette users. Multi-variable adjusted logistic regression was carried out using a backward deletion approach, starting with a full model of factors significantly associated with roll-your-own cigarette use at a 10% level of significance in the bivariate analysis. Statistical significance was set at p < 0.05 for the final model.

## Results

Current smoking tended to be lower in 2010 than during 2007 (18.1% vs. 20.9%; p = 0.07). Of the 1 296 current smokers in this study, 24.1% (n = 306) reported using roll-your-own cigarettes either exclusively, or with factory-made cigarettes (mixed users). Due to the small numbers of roll-your-own cigarette users in the white and Indian/Asian South African populations, these groups were combined for subsequent between-group analyses. Univariate analyses showed that roll-your-own cigarette use was highest among black African respondents (34.4%), and was more common among males than among females (27% vs 15.8%; p < 0.01). Roll-your-own cigarette use was highest among those with no education. Factory-made cigarette smoking was highest among those with 12 or more years of schooling (Table 1).

**Table 1:** Total sample characteristics and prevalence of use of any roll-your-own cigarette(s) compared to factory-made cigarettes among smokers

**Characteristics**		**% Total sample (N = 6019)**	**% Factory-made cigarettes only (N = 990)**	**% Any roll-your-own use (N = 306)**	p-value*
Survey year					0.02
	2007	48.6 (2907)	80.0 (520)	20.0 (156)	
	2010	51.4 (3112)	71.2 (470)	28.8 (150)	
Gender					0.00
	Male	48.2 (2489)	73.0 (600)	27.0 (223)	
	Female	51.8 (3530)	84.2 (390)	15.8 (83)	
Location					0.00
	Urban	66.5 (4257)	81.6 (818)	18.4 (158)	
	Rural	33.5 (1762)	60.9 (172)	39.1 (148)	
Age					0.35
	18-29	39.6 (1789)	74.8 (291)	25.2 (79)	
	30-40	23.1 (1481)	81.0 (268)	19.1 (75)	
	41-54	20.0 (1319)	73.4 (239)	26.6 (79)	
	>55	17.3 (1214)	72.7 (177)	27.3 (71)	
Education					0.00
	None	4.5 (303)	31.7 (20)	68.3 (47)	
	Grades 1-11	53.9 (3178)	71.2 (520)	28.8 (206)	
	≥Grade 12	41.6 (2467)	85.7 (447)	14.3 (52)	
Employment status					0.16
	Unemployed	31.2 (1504)	72.3 (218)	27.7 (82)	
	Student, Pensioner, permanently sick, housewife,	33.2 (2076)	72.7 (263)	27.3 (75)	
	Employed full or part time	35.6 (2297)	79.4 (497)	20.6 (148)	
Race					0.00
	Black African	76.7 (3593)	65.6 (359)	34.4 (214)	
	Coloured	9.4 (998)	86.4 (290)	13.6 (80)	
	Indian/White	14.0 (1427)	97.9 (341)	2.2 (12)	
					
Marital status					0.06
	Never married	55.5 (2763)	72.3 (406)	27.7 (158)	
	Widow/divorced/separated	11.0 (877)	83.2 (160)	16.8 (46)	
	Married	33.5 (2290)	79.0 (416)	21.0 (101)	
Current snuff use					0.37
	No	95.9 (5700)	76.2 (969)	23.8 (294)	
	Yes	4.2 (214)	66.5 (20)	33.5 (12)	
Total population			75.9 (95% CI: 72.1-79.3)	24.1 (95% CI: 20.7-27.9)	

*P-value from comparing group differences within any RYO use or factory-made cigarettes.

In 2007, 20% (n = 150; weighted population count = 1 289 345) of current smokers reported smoking roll-your-own cigarettes, but by 2010, 28.8% (n = 156; weighted population count = 1 658 407) of smokers reported using roll-your-own cigarettes (p = 0.02). Compared to those who exclusively smoked factory-made cigarettes, the roll-your-own cigarette smokers were more likely to be rural residents and uneducated. Those who did not plan to quit smoking tended to be more likely to use roll-your-own cigarettes than those who planned to quit (27.7% vs. 21.1%; p = 0.07). However, the use of roll-your-own cigarettes was not significantly associated with quit attempts or with having received any professional advice to quit or the existence of smoking rules at work or at home. In the final multi-variable adjusted model, it was clear that those who were more confident in their ability to successfully quit smoking were less likely to be roll-your-own cigarette smokers (OR = 0.72; 95% CI: 0.55-0.96). The final model also indicated that black South Africans were more likely to be roll-your-own cigarette smokers (OR = 20.13; 95% CI: 8.54-47.50) than white or Indian/Asian South Africans, independent of their level of education (Table 2).

**Table 2:** Logistic regression model of factors associated with smoking roll-your-own cigarettes

**Characteristics**		**Odds Ratio**	**95% Confidence Interval**
Education			
	≥Grade 12	1.0	
	Grades 1-11	1.79	1.13-2.83
	None	8.22	3.57-18.97
Location			
	Urban	1.0	
	Rural	1.63	1.02-2.61
Self-efficacy (per unit change)		0.72	0.55-0.96
Race			
	Indian/White	1.0	
	Coloured	5.53	2.23-13.69
	Black African	20.13	8.54-47.47
Survey year			
	2007	1.0	
	2010	1.24	1.07-1.44

NB: Predictor model significantly fits better than the null model [F df(7, 652) = 17.41; p < 0.001)].

## Discussion

This study demonstrates that despite an aggregate decline in the prevalence of smoking in South Africa, there has been a significant increase in the proportion of smokers in South Africa using roll-your-own cigarettes. These findings also suggest that there are currently about 1.7 million roll-your-own cigarette adult smokers in South Africa and that the majority live in rural areas. The greater use of roll-your-own cigarettes in the rural areas may in part reflect the fact that people living in rural areas are often poorer than their urban counterparts. The prevalence figures obtained in this study are higher than those reported for the US population [5]. The figures are consistent with the 23% reported for the UK [5], but are lower than the 50% reported for Thailand [11]. These findings also support a previous explanation offered, namely that the differences in the prevalence of the use of roll-your-own cigarettes across different countries may reflect the different ratios of urban: rural populations in the different countries, with Thailand having the highest rural population and the US with the lowest rural population [11].

This study also shows that the use of roll-your-own cigarettes was most common among people in the lower socio-economic group, in line with the findings in other studies [12]. The use of roll-your-own cigarettes was highest among black Africans, who are the poorest group in South Africa. Although poverty is not confined to any race group, in South Africa, it has historically been concentrated among black South Africans and coloured people (those of mixed descent) [13]. Taken together, this study’s findings suggest that it is likely that the use of roll-your-own cigarettes is partly motivated by the relatively high cost of factory-made cigarettes [1,5], considering that roll-your-own cigarettes are mostly smoked by people considered to be of low socio-economic status. In South Africa, those in the lower socio-economic group have been reported to be more sensitive to cigarette price increases [14], which is evidenced by a greater observed decrease in the consumption of factory-made cigarettes by this group [15]. The general increase in the use of roll-your-own cigarettes may nevertheless also be a result of the impact of the global financial crisis, which resulted in the loss of almost half a million jobs in South Africa during 2009 [16], which possibly then promoted the purchase of cheaper alternatives to factory-manufactured cigarettes by smokers who were unable to quit.

Tax increases are the most effective policy tool in reducing tobacco consumption, especially among the poor [17]. Higher taxes prevent smoking initiation, increase cessation among current users and eventually lead to substantial improvements in public health and lower social costs attributable to smoking [18]. However, these cigarette tax increases may also motivate some smokers to supplement factory-made cigarette smoking with roll-your-own cigarette smoking, or to switch completely to roll-your-own cigarettes as a cheaper alternative [19]. The observed increased use of roll-your-own cigarettes in South Africa from 2007 to 2010 may indeed negate the effect of increases in the price of factory-made cigarettes on smoking cessation - hence, there is a need for policy vigilance. In a study conducted in New Zealand, the relatively lower price of roll-your-own cigarette tobacco was the most commonly reported reason for smoking roll-your-own cigarettes, followed by other reasons such as the taste of roll-your-own cigarettes and a belief that these cigarettes are not as harmful as factory-made cigarettes [20]. Therefore, in addition to revising the tax structure to reduce the incentive to switch to pipe tobacco or roll-your-own cigarette tobacco [21], there is also a need to educate roll-your-own cigarette users on the dangers of using any tobacco product whatsoever.

As long as some cigarette users continue to regard roll-your-own cigarettes as a less harmful substitute for factory-made cigarettes, smokers may continue to change their smoking behaviour by opting for roll-your-own cigarettes, and they may not want to quit smoking roll-your-own cigarettes [20]. Although, roll-your-own cigarette smokers have been reported to be less likely to make quit attempts than those who smoke the more expensive factory-made cigarettes [8], we did not observe any difference in quit attempts between these two groups in the current study. This suggest that contrary to a prior observation that roll-your-own cigarette smokers are more addicted to smoking than other smokers [9], it may well be that roll-your-own cigarette smokers are as addicted to smoking as the smokers of factory-made cigarettes, but that roll-your-own smokers are less confident about being able to quit successfully, as is demonstrated by the findings of this study.

In developed countries such as the US and UK, lower socio-economic status has indeed been associated with low self-efficacy to quit, having no intention to quit and higher levels of nicotine dependence [22]. Hence, Siahpush et al. [22] suggest that population-level interventions such as tax and price increases may be less effective for lower social strata and that targeted interventions may be essential to reduce the disparities in cessation rates across socio-economic groups. This observation highlights the need for all healthcare providers to offer cessation assistance to all smokers, especially those of low socio-economic status. This is particularly important because this study has demonstrated that, despite having lower confidence in successfully quitting, roll-your-own cigarette smokers were no less likely to have attempted to quit smoking. Others have reported that quit rates are actually no different for roll-your-own cigarette smokers than for other smokers [23], which implies that with motivational support from health providers, a roll-your-own cigarette smoker is equally likely to succeed in a quit attempt, which in the long-term could reduce the growing burden of non-communicable diseases among low-income roll-your-own cigarettes smokers.

The findings of this study nevertheless need to be interpreted within the limitations of the study design. Firstly, this was a cross-sectional study; therefore inferences on causality should be made with caution, as there is no evidence of the temporal order of events. Secondly, the self-reported nature of the measures used may have also introduced reporting bias. However, considering that there is no evidence that reporting bias would be greater among a particular group of smokers, reporting bias is not very likely to be of concern in the present study. Furthermore, several studies have found that self-reporting is a valid means of assessing smoking status [24]; therefore the fact that self-reporting was used in this study is unlikely to have had a significant influence on the conclusions reached in this study. A major strength of this study lies in the use of a large nationally representative sample of all South Africans to explore the correlates of the use of an understudied tobacco product, namely roll-your-own cigarettes.

## Conclusion

This study found that the use of roll-your-own cigarettes is increasing in South Africa and that they are predominantly used by those in the lower socio-economic groups. Smokers who are not confident in quitting smoking successfully are more likely to be using roll-your-own cigarettes. From a policy perspective, higher taxes on roll-your-own cigarette products would discourage smoking in South Africa. From a clinical practice perspective, health providers would need to offer cessation assistance to all smokers, but particularly to those of low socio-economic status, in order to increase the effectiveness of tax policies in promoting tobacco use cessation.

## Endnotes

^a^ This classification is still used in the current South African population census to monitor progress in addressing racial inequality created by decades of apartheid rule. Prior to 1994, all people in South Africa were classified as black (people of indigenous African descent, who constitute 79% of the population), Asian/Indian (those of Indian or Asian ancestry), coloured (those of mixed ancestry) or white (those of European descent), according to the Population Registration Act of 1950. The use of these terms in the article and the survey in line with the census does not imply that the authors approve of this racist terminology or regard it as legitimate.

## Competing interests

The authors declare that they have no competing interests.

## Authors’ contributions

OAA conceived and designed the study, participated in the data collection, statistical analysis, and interpretation of the data and writing of the manuscript. BGO contributed to the data analysis and helped to draft the manuscript. Both authors read and approved the final version of the manuscript.

## Authors’ information

OAA is a public health practitioner and holds the position of Associate Professor of Health Promotion and Head of Clinical Unit at the Department of Community Dentistry at the University of Pretoria. OAA is also an Associate Professor in the School of Health Systems and Public Health at the University of Pretoria. BGO is an epidemiologist and a research assistant in the Department of Community Dentistry at the University of Pretoria.

## Pre-publication history

The pre-publication history for this paper can be accessed here:

http://www.biomedcentral.com/1471-2458/13/597/prepub
